# 2-Benzene­sulfonamido-3-methyl­butyric acid

**DOI:** 10.1107/S1600536812034393

**Published:** 2012-08-08

**Authors:** Muhammad Nadeem Arshad, Muhammad Danish, Muhammad Nawaz Tahir, Zain Ul Aabideen, Abdullah M. Asiri

**Affiliations:** a Center of Excellence for Advanced Materials Research (CEAMR), King Abdulaziz University, PO Box 80203, Jeddah 21589, Saudi Arabia; b Department of Chemistry, University of Gujrat, Gujrat 50781, Pakistan; c Department of Physics, University of Sargodha, Sargodha, Pakistan; d Department of Chemistry, Faculty of Science, King Abdulaziz University, PO Box 80203, Jeddah 21589, Saudi Arabia

## Abstract

In the crystal structure of the title compound, C_11_H_15_NO_4_S, two independent mol­ecules are present per asymmetric unit; they are dimerized through O—H⋯O hydrogen bonds between their carb­oxy groups to generate *R*_2_^2^(8) loops. An intra­molecular N—H⋯O link in one of the mol­ecules closes an *S*(5) ring. The dimers are linked by N—H⋯O and C—H⋯O hydrogen bonds to form a three-dimensional network. The C atoms of the isopropyl group of one of the mol­ecules are disordered over two orientations in a 3:1 ratio.

## Related literature

For biological studies of sulfonamides, see: Nalam *et al.* (2007 ▶). For related structures, see: Khan *et al.* (2011 ▶); Arshad *et al.* (2012 ▶). For graph-set notation, see: Bernstein *et al.* (1995 ▶).
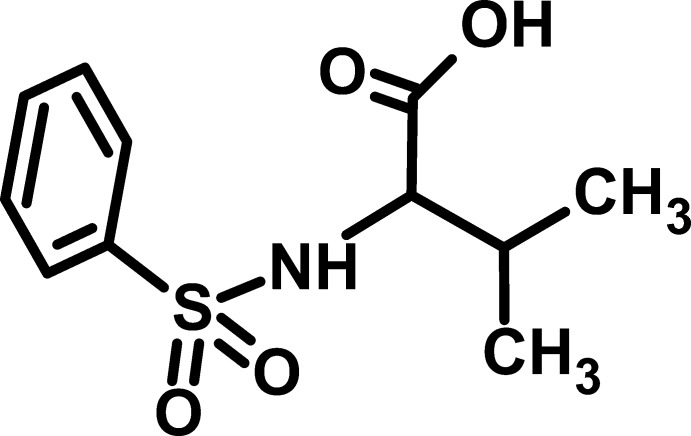


## Experimental

### 

#### Crystal data

C_11_H_15_NO_4_S
*M* *_r_* = 257.30
Monoclinic, 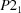

*a* = 5.4954 (3) Å
*b* = 15.5097 (10) Å
*c* = 15.5106 (9) Å
β = 94.043 (3)°
*V* = 1318.71 (14) Å^3^
*Z* = 4
Mo *K*α radiation
μ = 0.25 mm^−1^
*T* = 296 K
0.37 × 0.22 × 0.18 mm

#### Data collection

Bruker Kappa APEXII CCD diffractometer
Absorption correction: multi-scan (*SADABS*; Bruker, 2007 ▶) *T*_min_ = 0.914, *T*_max_ = 0.957
9573 measured reflections
4203 independent reflections
3803 reflections with *I* > 2σ(*I*)
*R*_int_ = 0.023

#### Refinement

*R*[*F* ^2^ > 2σ(*F* ^2^)] = 0.036
*wR*(*F* ^2^) = 0.093
*S* = 1.05
4203 reflections
335 parameters
7 restraints
H atoms treated by a mixture of independent and constrained refinement
Δρ_max_ = 0.18 e Å^−3^
Δρ_min_ = −0.18 e Å^−3^
Absolute structure: Flack (1983 ▶), 1785 Friedel pairs
Flack parameter: 0.00 (6)

### 

Data collection: *APEX2* (Bruker, 2007 ▶); cell refinement: *SAINT* (Bruker, 2007 ▶); data reduction: *SAINT*; program(s) used to solve structure: *SHELXS97* (Sheldrick, 2008 ▶); program(s) used to refine structure: *SHELXL97* (Sheldrick, 2008 ▶); molecular graphics: *PLATON* (Spek, 2009 ▶); software used to prepare material for publication: *WinGX* (Farrugia, 1999 ▶) and *X-SEED* (Barbour, 2001 ▶).

## Supplementary Material

Crystal structure: contains datablock(s) I, global. DOI: 10.1107/S1600536812034393/hb6922sup1.cif

Structure factors: contains datablock(s) I. DOI: 10.1107/S1600536812034393/hb6922Isup2.hkl

Supplementary material file. DOI: 10.1107/S1600536812034393/hb6922Isup3.cml

Additional supplementary materials:  crystallographic information; 3D view; checkCIF report

## Figures and Tables

**Table 1:** Hydrogen-bond geometry (Å, °)

*D*—H⋯*A*	*D*—H	H⋯*A*	*D*⋯*A*	*D*—H⋯*A*
O8—H8O⋯O3	0.85 (1)	1.81 (1)	2.649 (3)	171 (6)
O4—H4O⋯O7	0.86 (1)	1.83 (2)	2.668 (3)	166 (6)
N1—H1N⋯O1^i^	0.81 (5)	2.52 (5)	3.322 (3)	172 (5)
N2—H2N⋯O7^i^	0.82 (5)	2.47 (5)	3.240 (3)	157 (5)
N2—H2N⋯O8	0.82 (5)	2.45 (5)	2.734 (3)	102 (4)
C2—H2⋯O3^ii^	0.93	2.56	3.440 (4)	158
C4—H4⋯O6^iii^	0.93	2.54	3.427 (4)	161
C14—H14⋯O2^iv^	0.93	2.52	3.378 (5)	154
C15—H15⋯O1^v^	0.93	2.60	3.521 (5)	173
C9*A*—H9*A*⋯O2^v^	0.98	2.52	3.421 (7)	153

Symmetry codes: (i) 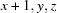; (ii) 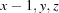; (iii) 
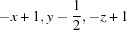
; (iv) 
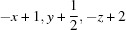
; (v) 
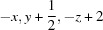
.

**Fig. 1.**
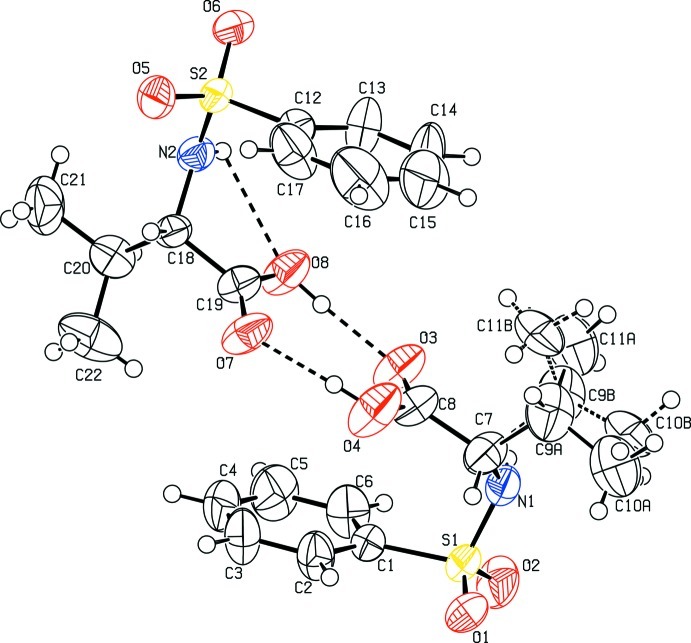
The labelled molecular structure of (I) with 50% displacement ellipsoids, showing intramolecular hydrogen bonding and formation of dimers through carboxylic group using dashed lines.

**Fig. 2.**
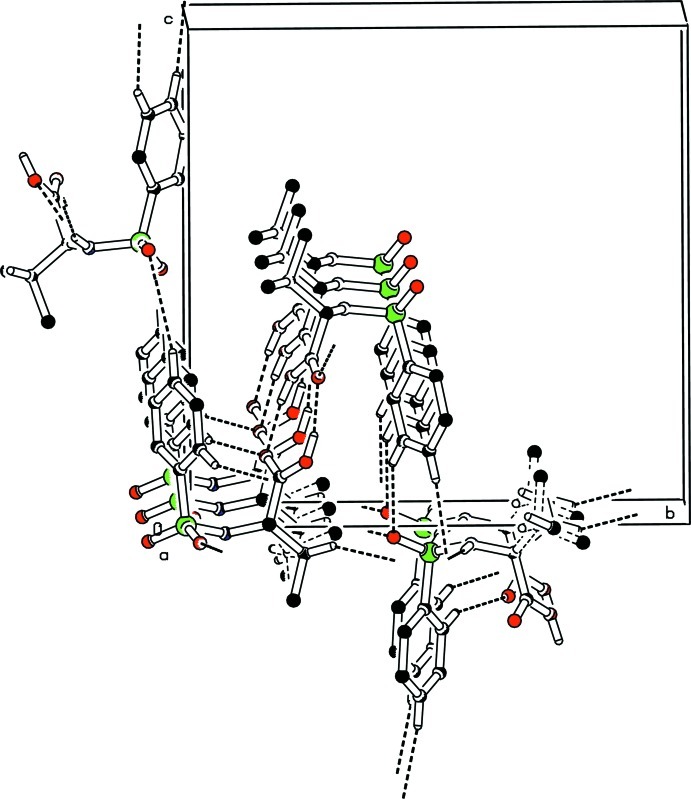
Unit cell packing showes intermolecular interactions using dashed lines.

## supplementary crystallographic information

### Comment

Crystal structure of sulfonamide derived from lysine has been reported which proved as a new class of inhibitors, to combat the resistant variants of HIV protease (Nalam *et al.*, 2007). Herein, we report the crystal structure of sulfonamide derived from L-valine, in countinuation of our work for the synthesis of sulfonamide (Khan *et al.*, 2011), (Arshad *et al.*, 2012).

The unit cell comprises of two independent molecules (C1—C11) A & (C12—C22) B. Each molecule adopted U shaped and aromatic rings are oriented with carboxylic groups at dihedral angles of 20.91 (2)° & 28.27 (2)° in molecule A and B respectively. These two molecules have been joined to each other through complex intermolecular hydrogen bonding interactions. Molecule B also undergoes intramolecular hydrogen bonding to form five membered (N2/C18/C19/O8/H2N) ring motif *S*_1_^1^(5) (Bernstein *et al.*, 1995). Molecules undergo typical dimerization to produce eight membered ring motifs *R*_2_^2^(8) (Bernstein *et al.*, 1995) through the carboxylic functional groups. This ring motif (O3/C8/O4/H4O/O7/C19/O8/H8O) lies between the two aromatic rings just like sandwich and the centroid distances of plane generated from it are 3.934 Å & 4.155 Å with aromatic rings (C1—C6) & (C12—C17) respectively. These dimers further connected through the N—H···O, O—H···O and C—H···O type interaction to form three dimensional network (Table. 1, Fig. 2). The C- atoms of isopropyl group in molecule A is disordered over two positions with occupancies ratio of 3:1.

### Experimental

L-Valine (0.50 g, 0.043 mmole) dissolved in 12-15 mL distilled water using sodium carbonate (1M) to a pH of 8–9. Benzenesulphonyl chloride (0.75 g, 0.043 mmole) added within 3–5 min. The pH was adjusted by sodium carbonate (1M). Disappearance of suspended benzenesulfonyl chloride gave indication of completion of reaction. Then, dilute HCl was added dropwise to result in a pH 2–3. The precipitate was filtered, washed with plenty of water and dried. Colourless prisms were obtained upon recrystalization from methanol solution.

### Refinement

All the C—H and H-atoms were positioned with idealized geometry with C—H = 0.93 Å for aromatic, C—H = 0.96 Å for methyl group and C—H = 0.98 Å for tertiary, and were refined using a riding model with *U*_iso_(H) = 1.2 *U*_eq_(C) for aromatic & tertiary and *U*_iso_(H) = 1.5 *U*_eq_(C) for methyl carbon atoms.

The N—H = 0.81–0.82 (5) and O—H = 0.85—0.86 (1) Å hydrogen atoms were located with difference map and were refined with *U*_iso_(H) = 1.2 *U*_eq_(N) and *U*_iso_(H) = 1.5 *U*_eq_(O).

Reaction does not affect the chirality of product, and the chirality is that of the reactant (L-Valine).

The atoms C9—C11 were disordered over two positions with the occupancies of 0.75 for C9A—C11A and 0.25 for C9B—C11B. The temperature factors of pairs of atoms were restrained to be identical.

### Crystal data

**Table:**

C_11_H_15_NO_4_S	*F*(000) = 544
*M**_r_* = 257.30	*D*_x_ = 1.296 Mg m^−^^3^
Monoclinic, *P*2_1_	Mo *K*α radiation, λ = 0.71073 Å
Hall symbol: P 2yb	Cell parameters from 4554 reflections
*a* = 5.4954 (3) Å	θ = 2.6–24.4°
*b* = 15.5097 (10) Å	µ = 0.25 mm^−^^1^
*c* = 15.5106 (9) Å	*T* = 296 K
β = 94.043 (3)°	Prismatic, colorless
*V* = 1318.71 (14) Å^3^	0.37 × 0.22 × 0.18 mm
*Z* = 4	

### Data collection

**Table:**

Bruker Kappa APEXII CCD diffractometer	4203 independent reflections
Radiation source: fine-focus sealed tube	3803 reflections with *I* > 2σ(*I*)
Graphite monochromator	*R*_int_ = 0.023
φ and ω scans	θ_max_ = 25.0°, θ_min_ = 1.9°
Absorption correction: multi-scan (*SADABS*; Bruker, 2007)	*h* = −5→6
*T*_min_ = 0.914, *T*_max_ = 0.957	*k* = −15→18
9573 measured reflections	*l* = −18→18

### Refinement

**Table:**

Refinement on *F*^2^	Hydrogen site location: inferred from neighbouring sites
Least-squares matrix: full	H atoms treated by a mixture of independent and constrained refinement
*R*[*F*^2^ > 2σ(*F*^2^)] = 0.036	*w* = 1/[σ^2^(*F*_o_^2^) + (0.0543*P*)^2^ + 0.0931*P*] where *P* = (*F*_o_^2^ + 2*F*_c_^2^)/3
*wR*(*F*^2^) = 0.093	(Δ/σ)_max_ < 0.001
*S* = 1.05	Δρ_max_ = 0.18 e Å^−^^3^
4203 reflections	Δρ_min_ = −0.18 e Å^−^^3^
335 parameters	Extinction correction: *SHELXL97* (Sheldrick, 2008), Fc^*^=kFc[1+0.001xFc^2^λ^3^/sin(2θ)]^-1/4^
7 restraints	Extinction coefficient: 0.0039 (11)
Primary atom site location: structure-invariant direct methods	Absolute structure: Flack (1983), 1785 Friedel pairs
Secondary atom site location: difference Fourier map	Flack parameter: 0.00 (6)

### Special details

**Table:**

Geometry. All esds (except the esd in the dihedral angle between two l.s. planes) are estimated using the full covariance matrix. The cell esds are taken into account individually in the estimation of esds in distances, angles and torsion angles; correlations between esds in cell parameters are only used when they are defined by crystal symmetry. An approximate (isotropic) treatment of cell esds is used for estimating esds involving l.s. planes.
Refinement. Refinement of F^2^ against ALL reflections. The weighted R-factor wR and goodness of fit S are based on F^2^, conventional R-factors R are based on F, with F set to zero for negative F^2^. The threshold expression of F^2^ > 2sigma(F^2^) is used only for calculating R-factors(gt) etc. and is not relevant to the choice of reflections for refinement. R-factors based on F^2^ are statistically about twice as large as those based on F, and R- factors based on ALL data will be even larger.

### Fractional atomic coordinates and isotropic or equivalent isotropic displacement parameters (Å^2^)

**Table:**

	*x*	*y*	*z*	*U*_iso_*/*U*_eq_	Occ. (<1)
S1	0.01018 (13)	0.47881 (5)	0.94705 (4)	0.0526 (2)	
S2	0.63466 (11)	0.90716 (5)	0.55510 (4)	0.04379 (18)	
O1	−0.2265 (4)	0.50382 (18)	0.97036 (13)	0.0746 (7)	
O2	0.1285 (5)	0.40489 (17)	0.98428 (14)	0.0812 (7)	
O3	0.3451 (4)	0.64351 (16)	0.82551 (15)	0.0762 (7)	
O4	0.0009 (5)	0.7191 (2)	0.82050 (16)	0.0896 (9)	
O5	0.4720 (4)	0.94893 (15)	0.49394 (12)	0.0615 (6)	
O6	0.8908 (3)	0.91930 (15)	0.55236 (12)	0.0583 (5)	
O7	0.0874 (3)	0.74580 (16)	0.65554 (14)	0.0683 (6)	
O8	0.4671 (4)	0.69767 (18)	0.67244 (14)	0.0713 (7)	
N1	0.1901 (4)	0.55941 (18)	0.97225 (14)	0.0519 (6)	
N2	0.5961 (4)	0.80431 (17)	0.54293 (14)	0.0470 (6)	
C1	−0.0023 (4)	0.4673 (2)	0.83346 (15)	0.0478 (7)	
C2	−0.1899 (5)	0.5045 (2)	0.78370 (19)	0.0642 (9)	
H2	−0.3146	0.5332	0.8094	0.077*	
C3	−0.1909 (7)	0.4988 (3)	0.6954 (2)	0.0783 (11)	
H3	−0.3168	0.5243	0.6611	0.094*	
C4	−0.0131 (7)	0.4572 (3)	0.6580 (2)	0.0799 (11)	
H4	−0.0170	0.4533	0.5980	0.096*	
C5	0.1747 (7)	0.4201 (3)	0.7076 (2)	0.0877 (13)	
H5	0.2987	0.3916	0.6814	0.105*	
C6	0.1802 (6)	0.4251 (3)	0.7962 (2)	0.0721 (10)	
H6	0.3070	0.4000	0.8303	0.087*	
C7	0.1146 (7)	0.6464 (2)	0.95050 (19)	0.0640 (8)	
H7	−0.0636	0.6460	0.9511	0.077*	
C8	0.1658 (6)	0.6696 (2)	0.8590 (2)	0.0598 (8)	
C9A	0.2016 (11)	0.7179 (4)	1.0149 (3)	0.0846 (16)	0.75
H9A	0.1325	0.7736	0.9960	0.102*	0.75
C9B	0.336 (5)	0.6908 (11)	1.0186 (10)	0.0846 (16)	0.25
H9B	0.4889	0.6580	1.0183	0.102*	0.25
C10A	0.1194 (16)	0.6948 (6)	1.1039 (4)	0.1206 (18)	0.75
H10A	0.1968	0.6422	1.1235	0.181*	0.75
H10B	−0.0544	0.6874	1.1004	0.181*	0.75
H10C	0.1642	0.7403	1.1439	0.181*	0.75
C10B	0.252 (4)	0.699 (2)	1.1091 (11)	0.132 (8)	0.25
H10D	0.3754	0.7273	1.1455	0.198*	0.25
H10E	0.2248	0.6420	1.1317	0.198*	0.25
H10F	0.1035	0.7311	1.1075	0.198*	0.25
C11A	0.4800 (14)	0.7213 (6)	1.0175 (5)	0.1206 (18)	0.75
H11A	0.5307	0.7257	0.9597	0.181*	0.75
H11B	0.5466	0.6698	1.0441	0.181*	0.75
H11C	0.5375	0.7706	1.0504	0.181*	0.75
C11B	0.366 (4)	0.7811 (13)	0.9824 (12)	0.132 (8)	0.25
H11D	0.4428	0.8176	1.0264	0.198*	0.25
H11E	0.2083	0.8041	0.9643	0.198*	0.25
H11F	0.4648	0.7787	0.9339	0.198*	0.25
C12	0.5515 (5)	0.93452 (19)	0.65875 (17)	0.0517 (7)	
C13	0.6936 (7)	0.9040 (3)	0.72930 (18)	0.0840 (11)	
H13	0.8328	0.8715	0.7219	0.101*	
C14	0.6242 (12)	0.9230 (4)	0.8109 (2)	0.1203 (18)	
H14	0.7154	0.9018	0.8591	0.144*	
C15	0.4279 (12)	0.9714 (5)	0.8216 (3)	0.135 (2)	
H15	0.3843	0.9836	0.8771	0.162*	
C16	0.2912 (8)	1.0028 (4)	0.7526 (4)	0.130 (2)	
H16	0.1549	1.0364	0.7613	0.156*	
C17	0.3527 (6)	0.9852 (3)	0.6681 (3)	0.0889 (12)	
H17	0.2615	1.0073	0.6203	0.107*	
C18	0.3522 (4)	0.76567 (19)	0.53994 (17)	0.0444 (6)	
H18	0.2335	0.8099	0.5203	0.053*	
C19	0.2898 (5)	0.7353 (2)	0.62866 (19)	0.0515 (7)	
C20	0.3346 (5)	0.6908 (2)	0.4741 (2)	0.0617 (8)	
H20	0.4612	0.6485	0.4915	0.074*	
C21	0.3830 (8)	0.7240 (3)	0.3847 (2)	0.0929 (13)	
H21A	0.3896	0.6763	0.3455	0.139*	
H21B	0.5357	0.7543	0.3875	0.139*	
H21C	0.2542	0.7624	0.3646	0.139*	
C22	0.0909 (7)	0.6464 (3)	0.4729 (3)	0.1032 (15)	
H22A	−0.0359	0.6867	0.4557	0.155*	
H22B	0.0657	0.6249	0.5296	0.155*	
H22C	0.0873	0.5993	0.4327	0.155*	
H1N	0.329 (8)	0.544 (4)	0.967 (3)	0.124*	
H2N	0.701 (8)	0.775 (4)	0.569 (3)	0.124*	
H4O	0.016 (10)	0.736 (4)	0.7683 (15)	0.155*	
H8O	0.423 (9)	0.676 (4)	0.719 (2)	0.155*	

### Atomic displacement parameters (Å^2^)

**Table:**

	*U*^11^	*U*^22^	*U*^33^	*U*^12^	*U*^13^	*U*^23^
S1	0.0641 (4)	0.0565 (5)	0.0374 (3)	−0.0096 (3)	0.0055 (3)	0.0090 (3)
S2	0.0456 (3)	0.0506 (4)	0.0350 (3)	−0.0056 (3)	0.0010 (2)	0.0099 (3)
O1	0.0674 (13)	0.110 (2)	0.0498 (11)	−0.0205 (12)	0.0258 (10)	−0.0023 (12)
O2	0.1254 (19)	0.0546 (14)	0.0609 (12)	−0.0055 (15)	−0.0131 (12)	0.0208 (12)
O3	0.0810 (14)	0.0791 (18)	0.0719 (14)	0.0184 (13)	0.0298 (12)	0.0299 (13)
O4	0.0912 (17)	0.104 (2)	0.0765 (16)	0.0295 (15)	0.0289 (14)	0.0358 (16)
O5	0.0675 (12)	0.0630 (14)	0.0517 (11)	−0.0038 (10)	−0.0127 (9)	0.0186 (9)
O6	0.0503 (10)	0.0710 (16)	0.0540 (10)	−0.0132 (10)	0.0062 (8)	0.0129 (10)
O7	0.0496 (11)	0.0854 (17)	0.0726 (14)	0.0144 (10)	0.0227 (10)	0.0315 (12)
O8	0.0566 (12)	0.0935 (19)	0.0656 (14)	0.0184 (12)	0.0166 (10)	0.0356 (13)
N1	0.0609 (13)	0.0558 (17)	0.0387 (11)	−0.0016 (12)	0.0024 (10)	0.0051 (11)
N2	0.0405 (11)	0.0574 (17)	0.0440 (12)	−0.0009 (10)	0.0088 (9)	0.0049 (11)
C1	0.0488 (14)	0.0562 (19)	0.0386 (13)	−0.0048 (13)	0.0049 (11)	−0.0002 (13)
C2	0.0537 (16)	0.091 (3)	0.0480 (15)	0.0123 (15)	0.0015 (13)	−0.0100 (15)
C3	0.082 (2)	0.105 (3)	0.0460 (16)	0.012 (2)	−0.0087 (15)	−0.0079 (18)
C4	0.091 (2)	0.104 (3)	0.0449 (16)	−0.009 (2)	0.0090 (17)	−0.0220 (18)
C5	0.083 (2)	0.111 (4)	0.071 (2)	0.014 (2)	0.0245 (19)	−0.028 (2)
C6	0.0671 (18)	0.084 (3)	0.0648 (19)	0.0208 (18)	0.0004 (15)	−0.0091 (17)
C7	0.084 (2)	0.055 (2)	0.0549 (17)	−0.0038 (16)	0.0198 (15)	0.0013 (15)
C8	0.0667 (19)	0.056 (2)	0.0592 (17)	0.0038 (15)	0.0203 (15)	0.0137 (15)
C9A	0.118 (5)	0.060 (4)	0.076 (3)	0.017 (3)	0.008 (3)	−0.017 (3)
C9B	0.118 (5)	0.060 (4)	0.076 (3)	0.017 (3)	0.008 (3)	−0.017 (3)
C10A	0.145 (5)	0.124 (5)	0.092 (3)	−0.026 (4)	0.001 (3)	−0.035 (3)
C10B	0.138 (15)	0.163 (17)	0.094 (9)	−0.098 (14)	0.000 (9)	−0.032 (10)
C11A	0.145 (5)	0.124 (5)	0.092 (3)	−0.026 (4)	0.001 (3)	−0.035 (3)
C11B	0.138 (15)	0.163 (17)	0.094 (9)	−0.098 (14)	0.000 (9)	−0.032 (10)
C12	0.0509 (15)	0.059 (2)	0.0456 (15)	−0.0092 (13)	0.0033 (12)	−0.0048 (13)
C13	0.111 (3)	0.100 (3)	0.0396 (15)	0.019 (3)	−0.0019 (16)	0.0004 (19)
C14	0.175 (5)	0.148 (5)	0.0382 (19)	−0.020 (4)	0.007 (2)	−0.016 (3)
C15	0.140 (4)	0.196 (7)	0.074 (3)	−0.052 (5)	0.044 (3)	−0.075 (4)
C16	0.076 (3)	0.196 (7)	0.122 (4)	−0.007 (3)	0.031 (3)	−0.090 (4)
C17	0.0591 (19)	0.120 (4)	0.086 (2)	0.007 (2)	−0.0036 (17)	−0.041 (3)
C18	0.0423 (13)	0.0456 (17)	0.0457 (14)	−0.0035 (12)	0.0057 (11)	0.0087 (12)
C19	0.0486 (15)	0.0491 (18)	0.0579 (17)	0.0040 (13)	0.0118 (13)	0.0099 (13)
C20	0.0570 (17)	0.055 (2)	0.072 (2)	0.0017 (15)	0.0021 (14)	−0.0047 (16)
C21	0.121 (3)	0.101 (4)	0.055 (2)	−0.006 (3)	−0.002 (2)	−0.023 (2)
C22	0.086 (3)	0.076 (3)	0.147 (4)	−0.022 (2)	0.004 (3)	−0.030 (3)

### Geometric parameters (Å, º)

**Table:**

S1—O2	1.420 (3)	C9B—C11B	1.522 (10)
S1—O1	1.428 (2)	C9B—H9B	0.9800
S1—N1	1.624 (3)	C10A—H10A	0.9600
S1—C1	1.767 (2)	C10A—H10B	0.9600
S2—O5	1.414 (2)	C10A—H10C	0.9600
S2—O6	1.4239 (19)	C10B—H10D	0.9600
S2—N2	1.619 (3)	C10B—H10E	0.9600
S2—C12	1.754 (3)	C10B—H10F	0.9600
O3—C8	1.215 (4)	C11A—H11A	0.9600
O4—C8	1.300 (4)	C11A—H11B	0.9600
O4—H4O	0.859 (10)	C11A—H11C	0.9600
O7—C19	1.226 (3)	C11B—H11D	0.9600
O8—C19	1.288 (3)	C11B—H11E	0.9600
O8—H8O	0.849 (10)	C11B—H11F	0.9600
N1—C7	1.445 (4)	C12—C17	1.362 (5)
N1—H1N	0.81 (5)	C12—C13	1.382 (4)
N2—C18	1.466 (3)	C13—C14	1.379 (6)
N2—H2N	0.82 (5)	C13—H13	0.9300
C1—C6	1.359 (4)	C14—C15	1.334 (8)
C1—C2	1.371 (4)	C14—H14	0.9300
C2—C3	1.373 (4)	C15—C16	1.354 (8)
C2—H2	0.9300	C15—H15	0.9300
C3—C4	1.338 (5)	C16—C17	1.403 (6)
C3—H3	0.9300	C16—H16	0.9300
C4—C5	1.370 (5)	C17—H17	0.9300
C4—H4	0.9300	C18—C19	1.516 (4)
C5—C6	1.375 (5)	C18—C20	1.545 (4)
C5—H5	0.9300	C18—H18	0.9800
C6—H6	0.9300	C20—C22	1.505 (5)
C7—C8	1.510 (4)	C20—C21	1.521 (5)
C7—C9A	1.545 (6)	C20—H20	0.9800
C7—C9B	1.70 (2)	C21—H21A	0.9600
C7—H7	0.9800	C21—H21B	0.9600
C9A—C10A	1.526 (7)	C21—H21C	0.9600
C9A—C11A	1.528 (7)	C22—H22A	0.9600
C9A—H9A	0.9800	C22—H22B	0.9600
C9B—C10B	1.513 (10)	C22—H22C	0.9600
			
O2—S1—O1	121.01 (16)	C10B—C9B—H9B	111.3
O2—S1—N1	105.66 (14)	C11B—C9B—H9B	111.3
O1—S1—N1	106.25 (15)	C7—C9B—H9B	111.3
O2—S1—C1	107.95 (15)	C9B—C10B—H10D	109.5
O1—S1—C1	107.93 (13)	C9B—C10B—H10E	109.5
N1—S1—C1	107.33 (12)	H10D—C10B—H10E	109.5
O5—S2—O6	119.94 (12)	C9B—C10B—H10F	109.5
O5—S2—N2	107.56 (13)	H10D—C10B—H10F	109.5
O6—S2—N2	104.39 (12)	H10E—C10B—H10F	109.5
O5—S2—C12	108.11 (14)	C9B—C11B—H11D	109.5
O6—S2—C12	108.50 (12)	C9B—C11B—H11E	109.5
N2—S2—C12	107.75 (13)	H11D—C11B—H11E	109.5
C8—O4—H4O	120 (4)	C9B—C11B—H11F	109.5
C19—O8—H8O	112 (4)	H11D—C11B—H11F	109.5
C7—N1—S1	120.2 (2)	H11E—C11B—H11F	109.5
C7—N1—H1N	121 (4)	C17—C12—C13	121.8 (3)
S1—N1—H1N	108 (4)	C17—C12—S2	120.0 (2)
C18—N2—S2	121.25 (18)	C13—C12—S2	118.3 (2)
C18—N2—H2N	114 (4)	C14—C13—C12	118.4 (4)
S2—N2—H2N	114 (4)	C14—C13—H13	120.8
C6—C1—C2	120.7 (3)	C12—C13—H13	120.8
C6—C1—S1	119.6 (2)	C15—C14—C13	120.8 (5)
C2—C1—S1	119.6 (2)	C15—C14—H14	119.6
C1—C2—C3	119.0 (3)	C13—C14—H14	119.6
C1—C2—H2	120.5	C14—C15—C16	120.8 (4)
C3—C2—H2	120.5	C14—C15—H15	119.6
C4—C3—C2	120.8 (3)	C16—C15—H15	119.6
C4—C3—H3	119.6	C15—C16—C17	120.8 (5)
C2—C3—H3	119.6	C15—C16—H16	119.6
C3—C4—C5	120.2 (3)	C17—C16—H16	119.6
C3—C4—H4	119.9	C12—C17—C16	117.3 (4)
C5—C4—H4	119.9	C12—C17—H17	121.3
C4—C5—C6	120.1 (3)	C16—C17—H17	121.3
C4—C5—H5	120.0	N2—C18—C19	111.3 (2)
C6—C5—H5	120.0	N2—C18—C20	110.1 (2)
C1—C6—C5	119.2 (3)	C19—C18—C20	111.0 (3)
C1—C6—H6	120.4	N2—C18—H18	108.1
C5—C6—H6	120.4	C19—C18—H18	108.1
N1—C7—C8	111.9 (3)	C20—C18—H18	108.1
N1—C7—C9A	116.7 (3)	O7—C19—O8	123.5 (3)
C8—C7—C9A	111.5 (3)	O7—C19—C18	122.4 (2)
N1—C7—C9B	93.0 (6)	O8—C19—C18	114.1 (2)
C8—C7—C9B	108.3 (6)	C22—C20—C21	110.9 (3)
C9A—C7—C9B	29.9 (6)	C22—C20—C18	111.5 (3)
N1—C7—H7	105.2	C21—C20—C18	109.9 (3)
C8—C7—H7	105.2	C22—C20—H20	108.1
C9A—C7—H7	105.2	C21—C20—H20	108.1
C9B—C7—H7	132.1	C18—C20—H20	108.1
O3—C8—O4	124.1 (3)	C20—C21—H21A	109.5
O3—C8—C7	122.2 (3)	C20—C21—H21B	109.5
O4—C8—C7	113.7 (3)	H21A—C21—H21B	109.5
C10A—C9A—C11A	110.1 (6)	C20—C21—H21C	109.5
C10A—C9A—C7	108.6 (5)	H21A—C21—H21C	109.5
C11A—C9A—C7	107.8 (5)	H21B—C21—H21C	109.5
C10A—C9A—H9A	110.1	C20—C22—H22A	109.5
C11A—C9A—H9A	110.1	C20—C22—H22B	109.5
C7—C9A—H9A	110.1	H22A—C22—H22B	109.5
C10B—C9B—C11B	108.4 (17)	C20—C22—H22C	109.5
C10B—C9B—C7	110.8 (17)	H22A—C22—H22C	109.5
C11B—C9B—C7	103.5 (14)	H22B—C22—H22C	109.5
			
O2—S1—N1—C7	−174.7 (2)	C8—C7—C9A—C11A	−66.9 (6)
O1—S1—N1—C7	−45.0 (2)	C9B—C7—C9A—C11A	22.7 (11)
C1—S1—N1—C7	70.3 (2)	N1—C7—C9B—C10B	−86.3 (18)
O5—S2—N2—C18	−50.0 (2)	C8—C7—C9B—C10B	159.4 (17)
O6—S2—N2—C18	−178.45 (19)	C9A—C7—C9B—C10B	58 (2)
C12—S2—N2—C18	66.3 (2)	N1—C7—C9B—C11B	157.6 (14)
O2—S1—C1—C6	−32.5 (3)	C8—C7—C9B—C11B	43.4 (15)
O1—S1—C1—C6	−164.8 (3)	C9A—C7—C9B—C11B	−58.1 (13)
N1—S1—C1—C6	81.0 (3)	O5—S2—C12—C17	2.4 (3)
O2—S1—C1—C2	150.6 (3)	O6—S2—C12—C17	134.0 (3)
O1—S1—C1—C2	18.3 (3)	N2—S2—C12—C17	−113.6 (3)
N1—S1—C1—C2	−95.9 (3)	O5—S2—C12—C13	−177.0 (3)
C6—C1—C2—C3	−0.2 (6)	O6—S2—C12—C13	−45.4 (3)
S1—C1—C2—C3	176.7 (3)	N2—S2—C12—C13	67.0 (3)
C1—C2—C3—C4	0.5 (6)	C17—C12—C13—C14	2.7 (7)
C2—C3—C4—C5	−0.7 (7)	S2—C12—C13—C14	−177.9 (4)
C3—C4—C5—C6	0.5 (7)	C12—C13—C14—C15	−1.6 (8)
C2—C1—C6—C5	0.0 (6)	C13—C14—C15—C16	0.3 (10)
S1—C1—C6—C5	−176.9 (3)	C14—C15—C16—C17	0.0 (10)
C4—C5—C6—C1	−0.2 (7)	C13—C12—C17—C16	−2.5 (6)
S1—N1—C7—C8	−84.3 (3)	S2—C12—C17—C16	178.1 (4)
S1—N1—C7—C9A	145.6 (3)	C15—C16—C17—C12	1.1 (8)
S1—N1—C7—C9B	164.6 (6)	S2—N2—C18—C19	−93.4 (2)
N1—C7—C8—O3	−35.6 (5)	S2—N2—C18—C20	143.0 (2)
C9A—C7—C8—O3	97.1 (5)	N2—C18—C19—O7	138.6 (3)
C9B—C7—C8—O3	65.4 (8)	C20—C18—C19—O7	−98.4 (3)
N1—C7—C8—O4	144.1 (3)	N2—C18—C19—O8	−41.7 (4)
C9A—C7—C8—O4	−83.3 (4)	C20—C18—C19—O8	81.3 (3)
C9B—C7—C8—O4	−114.9 (7)	N2—C18—C20—C22	176.9 (3)
N1—C7—C9A—C10A	−55.9 (7)	C19—C18—C20—C22	53.2 (4)
C8—C7—C9A—C10A	173.9 (5)	N2—C18—C20—C21	−59.7 (3)
C9B—C7—C9A—C10A	−96.6 (14)	C19—C18—C20—C21	176.6 (3)
N1—C7—C9A—C11A	63.4 (6)		

### Hydrogen-bond geometry (Å, º)

**Table:**

*D*—H···*A*	*D*—H	H···*A*	*D*···*A*	*D*—H···*A*
O8—H8*O*···O3	0.85 (1)	1.81 (1)	2.649 (3)	171 (6)
O4—H4*O*···O7	0.86 (1)	1.83 (2)	2.668 (3)	166 (6)
N1—H1*N*···O1^i^	0.81 (5)	2.52 (5)	3.322 (3)	172 (5)
N2—H2*N*···O7^i^	0.82 (5)	2.47 (5)	3.240 (3)	157 (5)
N2—H2*N*···O8	0.82 (5)	2.45 (5)	2.734 (3)	102 (4)
C2—H2···O3^ii^	0.93	2.56	3.440 (4)	158
C4—H4···O6^iii^	0.93	2.54	3.427 (4)	161
C14—H14···O2^iv^	0.93	2.52	3.378 (5)	154
C15—H15···O1^v^	0.93	2.60	3.521 (5)	173
C9*A*—H9*A*···O2^v^	0.98	2.52	3.421 (7)	153

Symmetry codes: (i) *x*+1, *y*, *z*; (ii) *x*−1, *y*, *z*; (iii) −*x*+1, *y*−1/2, −*z*+1; (iv) −*x*+1, *y*+1/2, −*z*+2; (v) −*x*, *y*+1/2, −*z*+2.
